# Sustainable grafted chitosan-dialdehyde cellulose with high adsorption capacity of heavy metal

**DOI:** 10.1186/s13065-023-01035-9

**Published:** 2023-09-20

**Authors:** Essam S. Abd El-Sayed, Sawsan Dacrory, Hisham A. Essawy, Hanan S. Ibrahim, Nabila S. Ammar, Samir Kamel

**Affiliations:** 1 Cellulose and Paper Department, National Research Centre, Dokki, 12622 Cairo Egypt; 2 Department of Polymers and Pigments, National Research Centre, Dokki, 12622 Cairo Egypt; 3 Department of Water Pollution, National Research Centre, Dokki, 12622 Cairo Egypt

**Keywords:** Dialdehyde Cellulose, Chitosan, Grafting, Heavy Metal Ions Removal

## Abstract

A novel adsorbent was prepared using a backbone comprising chemically hybridized dialdehyde cellulose (DAC) with chitosan via Schiff base reaction, followed by graft copolymerization of acrylic acid. Fourier transform infrared spectroscopy (FTIR) confirmed the hybridization while scanning electron microscopy (SEM) revealed intensive covering of chitosan onto the surface of DAC. At the same time, energy dispersive X-ray (EDX) proved the emergence of nitrogen derived from chitosan. The X-ray diffraction (XRD) indicated that the crystallinity of the backbone and graft copolymer structures was neither affected post the hybridization nor the grafting polymerization. The adsorbent showed high swelling capacity (872%) and highly efficient removal and selectivity of Ni^2+^ in the presence of other disturbing ions such as Pb^2+^ or Cu^2+^. The kinetic study found that the second-order kinetic model could better describe the adsorption process of (Cu^2+^, Ni^2+^) on the graft copolymer. In contrast, the first-order kinetic model prevails for the binary mixture (Pb^2+^, Ni^2+^). Moreover, the correlation coefficient values for the adsorption process of these binary elements using Langmuir and Freundlich isotherms confirmed that the developed grafted DAC/chitosan exhibits a good fit with both isotherm models, which indicates its broadened and complicated structure. Furthermore, the grafted DAC/chitosan exhibited high efficient regeneration and high adsorption capacity for Pb^2+^, Cu^2+^ and Ni^2+^.

## Introduction

Chitosan is a material from crustacean shells and is usually wasted in seafood industries. Therefore, it can be applied as a suitable biopolymer in manufacturing blended or composite materials [1–3]. Chitosan possesses a cycloaliphatic structure that comprises both active amino and hydroxyl groups. Due to its inferior mechanical properties, chitosan can be crosslinked by several reagents, among which are glutaraldehyde [4–6], formaldehyde [7, 8], and sulfuric acid [9, 10]. Because it has numerous chelation sites, amino and hydroxyl groups that adsorb the metal ions via coordination bonds or ion exchange, and multiple chelation sites, chitosan is a great choice for adsorbing the hazardous metal ions from an aqueous solution. Therefore, numerous researchers have looked at how chitosan and its modified forms' capacity for adsorption can be employed to remove different heavy metals [11]. In one attempt, He et al. studied the removal of Cu^2+^ and Ni^2+^ by amidoxime-functionalized chitosan, and it was found that the adsorption capacity was a function of initial concentration, pH solution, coexisting ions, and process temperature [12]. The effect of coexisting cations, including Na^+^, Ca^2+^, and Fe^3+^, on the adsorption of Cu^2+^ onto chitosan-grafted maleic acid composite was clarified by another investigation. It was found that the presence of Fe^3+^ significantly impacted the efficiency of the adsorption process. This effect may be attributed to the development of hydroxyl-Fe-coated chitosan-grafted maleic acid that intercalates Cu^2+^. The rivalry between Cu^2+^ and the interfering cations (Na^+^ and Ca^2+^) for the negative adsorption sited on the surface of chitosan-grafted maleic acid, on the other hand, produced a reduction in the Cu^2+^ adsorption capacity. Additionally, the shielding of Cu^2+^ caused by the increase in cations near the composite surface lowers the effectiveness of adsorption [13].

Further, cellulose, the most widely available organic biopolymer in nature, is traditionally a high molecular weight complicated structure with long chains of d-glucose units linked with each other via β-1,4-glucosidic bonds. It is non-toxic, renewable, biodegradable, and modifiable to have great potential as an excellent industrial material [7, 14]. The excellent properties of cellulose are motivating researchers to undergo intensive efforts as a step on the way to develop novel hybridized biopolymeric structures with outstanding properties, especially by combining/blending with other materials to remove organic and inorganic pollutants from wastewater [15–18]. Among the different available forms of cellulose, oxidized cellulose is the functionalized cellulose derivative with high reactivity and is nontoxic, biodegradable, and biocompatible. Selective cellulose oxidation is a robust method of modification to give high-performance cellulose-based materials for many applications, including environmental, energy, smart materials, biomedical engineering and healthcare, barrier applications, and active food packaging [19]. Periodate oxidation of cellulose is a reaction system that cleaves the C2–C3 bonds in monomer units and oxidizes the vicinal hydroxyl groups into 2,3 dialdehyde moieties, dialdehyde cellulose (DAC). The advantage of this process is the possibility of further dialdehyde oxidation into carboxylic ones [20, 21].

Moreover, its hydrophilicity, which the copious hydroxyl groups provide, makes it attractive to many researchers [22]. A material with such exciting features can greatly provoke a dramatic change in the emergence of highly valued materials for different applications. Functionalization of DAC can expand its applications' fields and make it easier to be involved in constructing of crosslinked network structures. This is a promising way to obtain new soft materials with superior properties and multifunctional ability [23].

Interestingly, the high aspect ratio of DAC renders it more liable and easier for efficient modification, especially in the presence of a huge number of hydroxyl groups on the surface, presenting a unique functionalization platform [23]. This is mainly achieved by conferring new groups on the surface while keeping the integrity of the crystalline core unchanged [24, 25].

Among the significant uses of chitosan and cellulose is their employment as backbones for grafting polymerization [26–28]. Cellulose was combined previously with chitosan using different linking agents such as urea–formaldehyde-sulphuric acid [28], 3-methyl glutaric anhydride [29], and Schiff base crosslinking [30]. Furthermore, the applicability of chitosan-cellulose hybrids as precursors for the fabrication of superabsorbent materials was presented before [30–33].

In continuation of these efforts, superabsorbent materials were prepared via the grafting polymerization of acrylic acid on a backbone incorporating dialdehyde cellulose (DAC), chemically hybridized with chitosan. The potential of the resulting material to reveal remarkable sorption selectivity during the adsorption of heavy metal ions with known removal difficulty, such as nickel, will be explored in the current work, particularly in the presence of other confusing ions like lead and copper. Also, kinetics and isotherms were studied to understand the mechanism of adsorption.

## Materials and methods

### Materials

Bagasse bleached pulp was obtained from Quena Company of Paper Industry, Egypt, as a raw material. The chemical composition of the pulp was defined according to Tappi standards with cellulose content (96%), hemicellulose (3%), and very low lignin content. Chitosan, with a deacetylation degree > 90%, was supplied from Oxford Laboratory, India. Sodium periodate was obtained from Analytical Rasayan, India. Acrylic acid was provided from Sigma-Aldrich, USA. In addition, N,N-methylene bisacrylamide (MBA) was ordered from Acros Organics, USA. All other chemicals in this study were analytical grade and used without further purification.

### Preparation of dialdehyde cellulose (DAC)

The dialdehyde cellulose (DAC) was prepared using a previously described procedure [34]. Briefly, 10 g cellulose was poured into 100 mL of deionized water and stirred to get a homogenized suspension. Then, 16 g of sodium periodate was added to the cellulose suspension, the pH was adjusted to 3 using 1 M sulfuric acid solution, and the stirring continued in darkness at room temperature for 24 h. Next, excess ethylene glycol was added to quench the reaction and ensure the decomposition of the remaining periodate. The oxidized product, DAC, was precipitated by pouring the solution into a significant excess of ethanol, which was washed several times alternatively with ethanol and water [35]. Finally, the product was filtrated and dried in an oven overnight at 60 °C.

### Preparation of DAC/chitosan hybrid

Chitosan (5 g) was dissolved in a 125 mL hydrochloric acid solution (0.5 M). Different weight ratios of DAC to chitosan (1:1, 1:2, and 1:3wt/wt) were employed and stirred at 50 °C for 2 h afterward; the resulting slurry was washed several times using ethanol before drying at room temperature [34].

### Preparation of superabsorbent

As the initial step for preparing a superabsorbent, the DAC/chitosan hybrid, formulated with 1:2, was selected as a backbone for completing the grafting polymerization of acrylic acid due to its superior handling concerning the other ratios in the sense that it was coherent and not brittle due to the reinforced structure. Thus, the superabsorbent was prepared by charging various weight ratios of acrylic acid: 3:1, 6:1, and 9:1 with respect to the DAC/chitosan hybrid. The mixture was dispersed in distilled water in a round flask, followed by the addition of potassium persulphate (KPS) as an initiator (0.2 g) and methylene bisacrylamide (MBA) as a crosslinker (0.1 g). The reaction mixture was kept in a water bath at 60 °C under continuous stirring at 500 rpm for 1 h. Next, the produced graft copolymers, symbolized as **H1, H2,** and **H3**, were washed with excess water and then used ethanol to remove the excess water [36]. Finally, the grafted products were dried at 60 °C in the oven. The grafting percentage (G %) was calculated by the following equations:

$$\mathrm{G\%}=\frac{Wf-Wi}{Wi}X 100$$

where *W*_*f*_ and *W*_*i*_ are the weight of the DAC/chitosan after and before the grafting polymerization, respectively.

The G% of H1, H2, and H3 were 235, 273, and 301, respectively.

### Characterizations

Fourier transform infrared spectroscopy (FTIR) was performed on KBr discs of the samples using a Shimadzu 8400 S FT-IR spectrophotometer, and the relevant spectra were recorded in the range of 400–4000 cm^−1^. X-ray diffractograms were collected for the samples at 25 °C using a Diano X-ray diffractometer (XRD) equipped with a monochromatic Cu Kα radiation source (λ = 0.154 nm, 2θ = 5: 70°) over a scanning time of 5 min. An environmental scanning electron microscope (SEM), Quanta-250 fitted with EDX unit, FEI IN SPECTS Company, Philips, Holland, was used to examine the different samples' morphological aspects without coating.

### Water uptake

The water uptake of the formed superabsorbent was determined gravimetrically using a dry piece by immersion in distilled water for 24 h to allow complete swelling at room temperature. The swollen piece was removed and wiped gently between two filter papers. The water uptake (%) was then calculated as an average of three measurements using the following equation [37]:

$$\mathrm{Water\, Uptake \%}=\frac{\left(\mathrm{M}2-\mathrm{M}1\right)}{\mathrm{M}1}\mathrm{ X }100$$

where; M_1_ and M_2_ are the dry and wet weights of the superabsorbent, respectively.

### Adsorption of heavy metal ions

Batch adsorption experiments were performed by mixing 0.1–0.15 g of the superabsorbent with 100 mL of binary solutions, pre-adjusted at pH 5.6, incorporating 10 mg/L for each element of the metal ions; (Pb^2+^/Ni^2+^) and (Cu^2+^/Ni^2+^). The binary systems were shaken at 150 rpm for 120 min at room temperature. After that, the mixtures were filtered using filter papers (Whatman^®^, No. 3). Then, the metal ions concentration was determined in the filtrate by inductive coupled plasma optical emission spectrometry (Agilent ICP-OES 5100, Australia) according to a standard method, designed for examination of water/wastewater [38]. Wavelength and operating conditions were; RF power = 1.3 kW—Plasma gas flow = 15 Lmin^−1^—Auxiliary Ar, 0.2 Lmin^−1^—Nebulizer Ar, 0.8 Lmin^−1^—Pump rate, 1.5 mLmin^−1^—Readings/replicate 3—Wavelength, Cu, Ni, and Pb 324.752, 231.604, and 220.353 nm, respectively. The presented data represent the average value of three readings.

### Adsorption kinetics and isotherms

The kinetics and isotherms statistics assessment is crucial to explore the mechanism of the sorption reaction and the type of interaction between the sorbate and adsorbent, respectively. Therefore, the adsorption kinetics was studied using pseudo-first-order and pseudo-second-order, while the adsorption isotherms were controlled using Langmuir and Freundlich models [39].

#### Desorption studies

The typical batch sorption process of 0.1 g of sorbent in 100 mL of 10 mg/L binary solution (Pb^+2^, Ni^+2^) and (Cu^+2^, Ni^+2^) was operated at the optimum operating condition. Then, the mixture was filtered through filter paper (No. 41), and the filtrate concentration was analyzed. The loaded sorbent was placed in 100 mL of 0.1 M HCl and stirred for 2 h to elution the ions from the sorbent, (Pb^+2^, Ni^+2^) and (Cu^+2^, Ni^+2^) ions at room temperature. After filtration, the sorbent was washed with deionized water; the pH was adjusted using 0.1 M KOH to 6, filtered, washed with deionized water then the sorbent was employed for the next cycle.

## Results and discussion

**Scheme 1:**
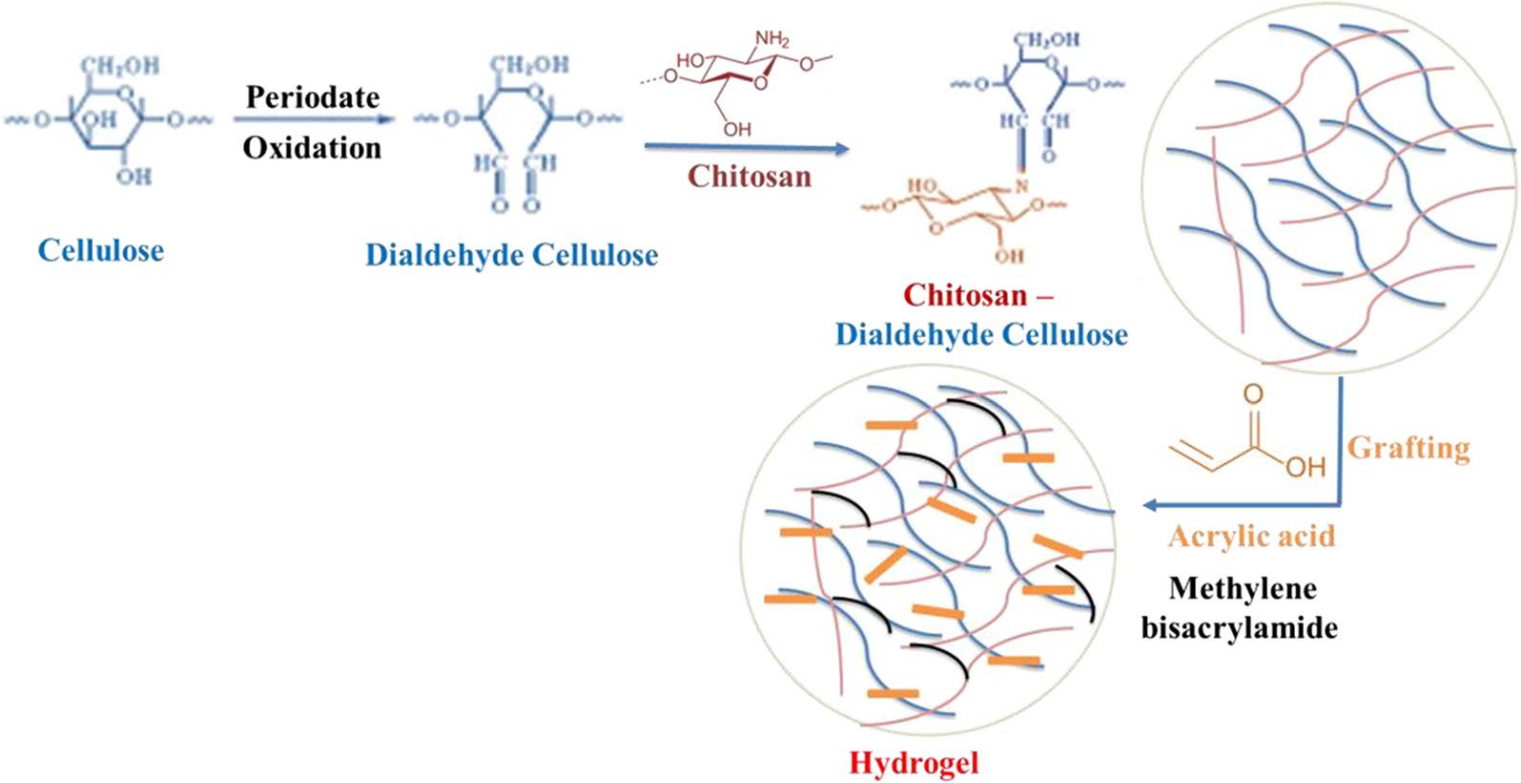
Schematic representation of superabsorbent hydrogel

Cellulose was surface-functionalized and offered high adsorption efficiency toward different heavy metals. As a result of its ability to target the vicinal secondary hydroxyl groups at C2 and C3 and cleave the C2-C3 bond in the anhydroglucose structural unit, sodium periodate is a highly selective cellulose oxidant that produces two vicinal aldehyde groups (dialdehyde cellulose, DAC). The procedure occurs in the dark, at an acidic pH of 3, and at temperatures close to room temperature [35]. Accordingly, the present study proposes a novel route to synthesize highly metal-adsorbed hydrogel (Scheme 1). Specifically, cellulose was oxidized by NaIO_4_to yield DAC [40]. The Schiff-base reaction between the dialdehyde groups on oxidized cellulose and the glucosamine groups of chitosan leads to the formation of the crosslinking network. Additionally, the superabsorbent hydrogel was formed by the radical polymerization reaction using potassium persulfate as an initiator, which is soluble in water and does not form alkoxide radicals, so it is the best radical initiator for hydrogen abstraction [41]. It was used for hydrogen abstraction of the primary OH groups in cellulose and/or chitosan transformed into radicals which initiated graft polymerization of acrylic acid onto DAC/Chitosan.

### Structural characterization of the hydrogel

#### FT-IR analysis

**Fig. 1:**
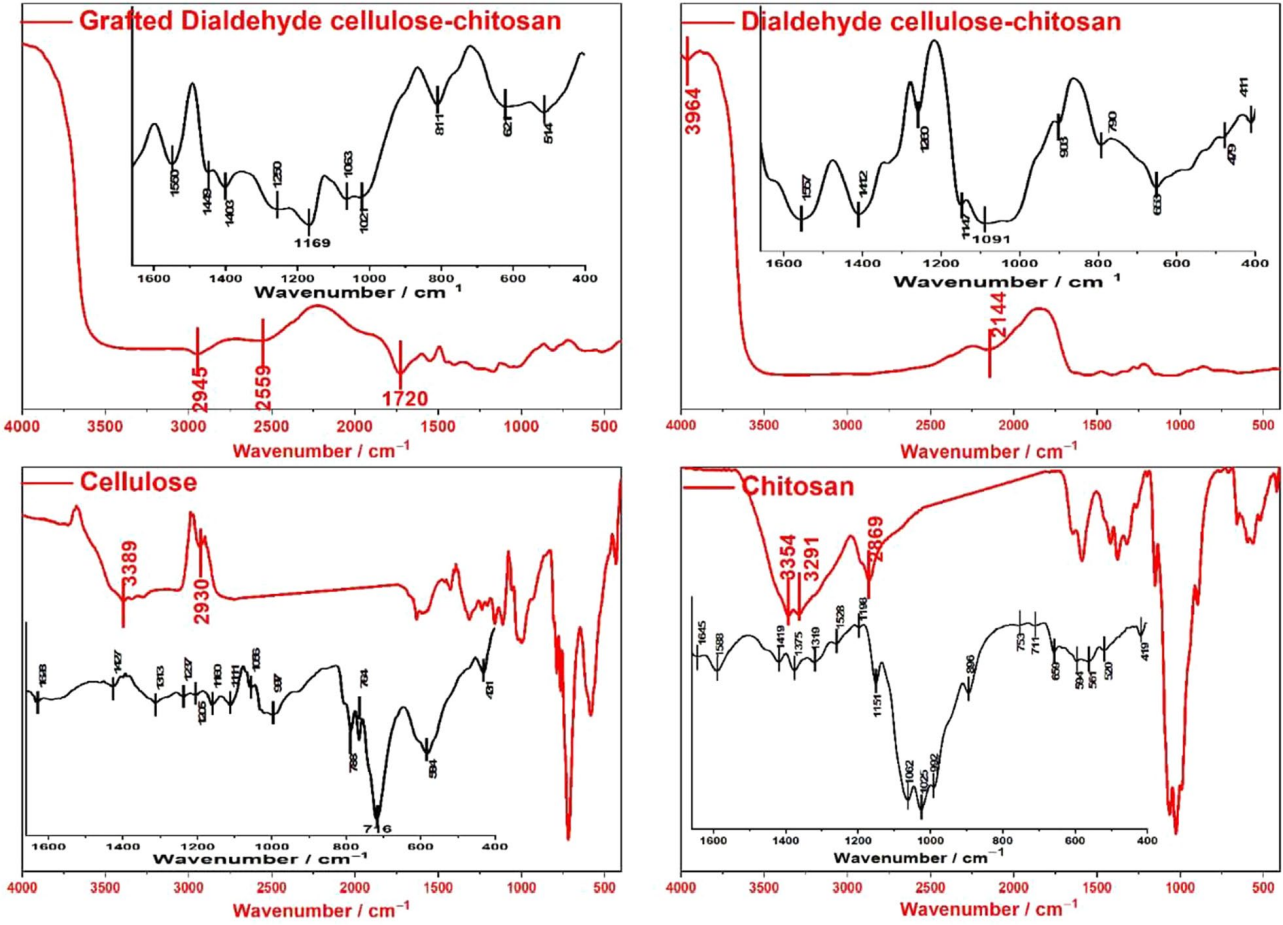
FTIR spectra of; cellulose, chitosan, DAC/chitosan, and grafted DAC/chitosan

The FTIR spectrum of the prepared cellulose nanofibers is presented in Fig. 1. As expected, the bands at 3450–3389 and 1021 cm^−1^ are dominant and refer to the stretching vibrations of the OH and C-O groups, respectively. In addition, the OH bending appears in the 1520–1648 cm^−1^ region. The broadness of this band can be justified by the adsorbed water, which is known to be strongly interacting with cellulosic chains. The peaks at 2826 and 2960 cm^−1^ represent asymmetric and symmetric vibrations of CH_2_ groups, while the peak at 1370–1395 cm^−1^ represents the scissoring motion of CH_2_ [31]. The band of C–O–C, which indicates the pyranose ring stretching vibration in cellulose, is centered at 1048 cm^−1^, while another peak, centered at 788 cm^−1^, is ascribed to the β-glycosidic linkages of cellulose. Also, the peak at 1130 cm^−1^ presents the C–C ring stretching. The absence of a distinct characteristic peak around 1720–1740 cm^−1^ confirms that all the lignin and hemicellulose have gone through successive chemical treatments [42].

The spectrum of DAC shows the emergence of an influential band at 1740 cm^−1^, corresponding to the insertion of the aldehydic carbonyl groups. Interestingly, this emergence is associated with the broadening and shift of the band centered at 1642 cm^−1^ along with complete flattening and collapse of the band in the region around 3340–3555 cm^−1^, which confirms that aldehyde group insertion took place on the OH groups [43].

The relevant FTIR spectrum of chitosan shows a broad overlapping peaks at 3354 and 3291 cm^–1^, allotting the OH and NH_2_ frequencies of chitosan, together with a peak at 1638 cm^–1^, assigning the amidic C = O and the primary NH_2_ of chitosan. The C–O stretching of the primary alcoholic groups is revealed by a significant band at 1375 cm^–1^. Also, a peak at 1025 cm^–1^ corresponding to amino groups of glucosamine units can be observed together with a peak at 896 cm^–1^ ascribed to C–N stretching. The absorption peak at 1063 cm^–1^ is attributed to C–O glucose bending, while those at 1419 and 1476 cm^–1^ describe the C–H bending of—CH_2_OH [32].

The DAC-chitosan hybrid is characterized by a fall down of the aldehydic carbonyl intensity and almost disappearance of the band at 1638 cm^–1^, which is assigned to the primary NH_2_ of chitosan. This is evidence that the hybridization proceeds principally via a Schiff base reaction between the aldehyde groups of DAC and amino groups of the chitosan. In addition, this can be further corroborated by the small peaks at 1412 and 2144 cm^–1^, which refer to C-N and C = N bonds, respectively. Moreover, the ill-identified nature of the band beyond 3000 cm^–1^ further supports this assumption. Finally, it is worth noting broadband extended from 1640 to 2140 cm^–1^, which is thought to combine the overlapping of many carbonyl environments and C = C derived from lignin and/or hemicellulose. This also reveals these components' participation in the hybridization process with chitosan.

The graft copolymerization of acrylic acid on the DAC-chitosan backbone yielded an unequivocal band at 2945 cm^−1^, attributed to the asymmetric and symmetric vibrations of CH_2_ groups from the incorporated acrylic acid. In contrast, the carboxyl groups of acrylic acid are recognized by the appearance of a band at 1720 cm^–1^ and a broadened band around 2515–2748 cm^−1^ [44].

#### XRD investigation on the prepared superabsorbent

**Fig. 2:**
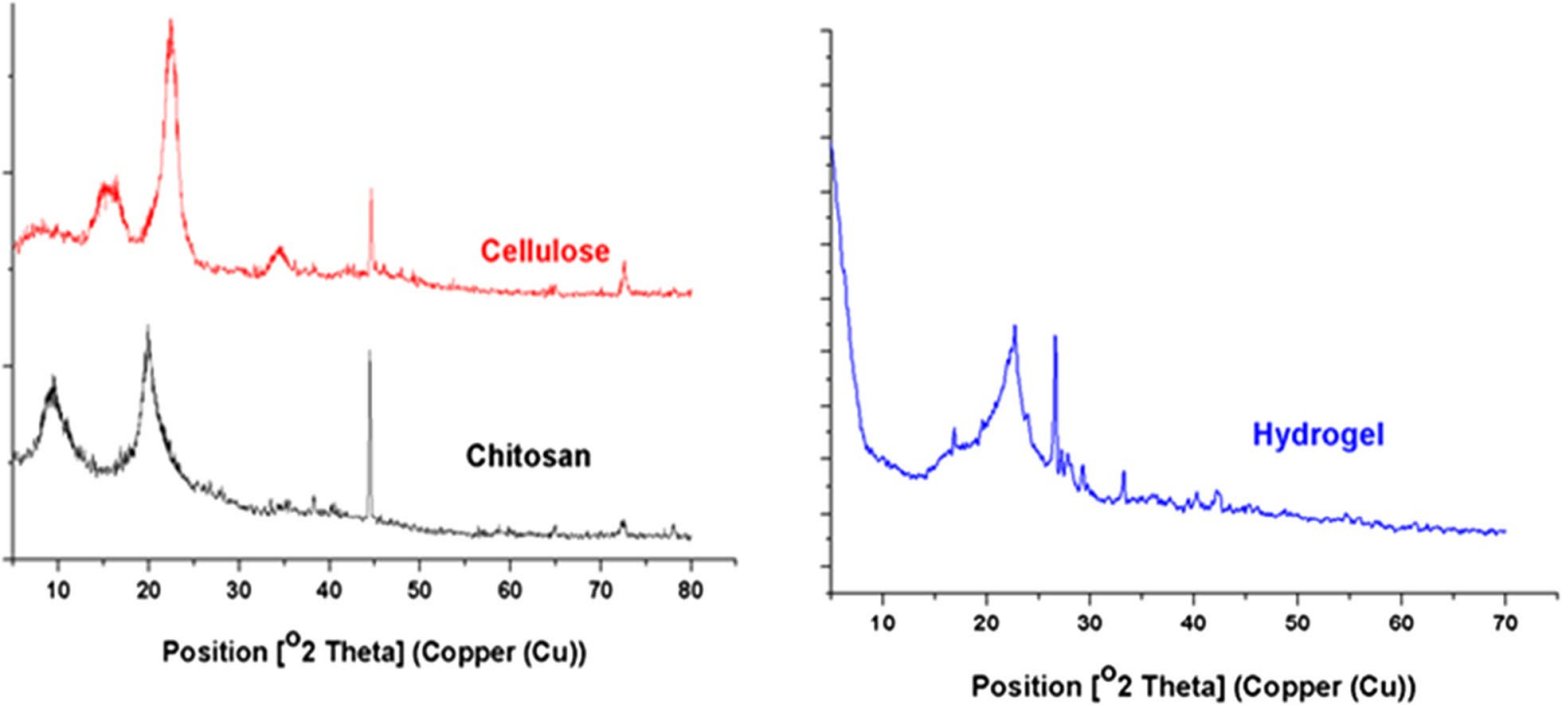
XRD spectra of; cellulose, chitosan, and grafted DAC/chitosan

X-ray diffraction (XRD) was performed to check the crystallinity of cellulose, chitosan, and grafted DAC/chitosan (Fig. 2). Several diffractions can be observed at 14.9°, 21.97°, and 26.5°, which are typical of both chitosan and DAC [32]. These patterns indicate that neither the chemical hybridization between DAC and chitosan nor the graft copolymerization caused alteration of the crystalline skeleton of the main components.

#### Surface morphology and elemental composition for the hydrogels

**Fig. 3:**
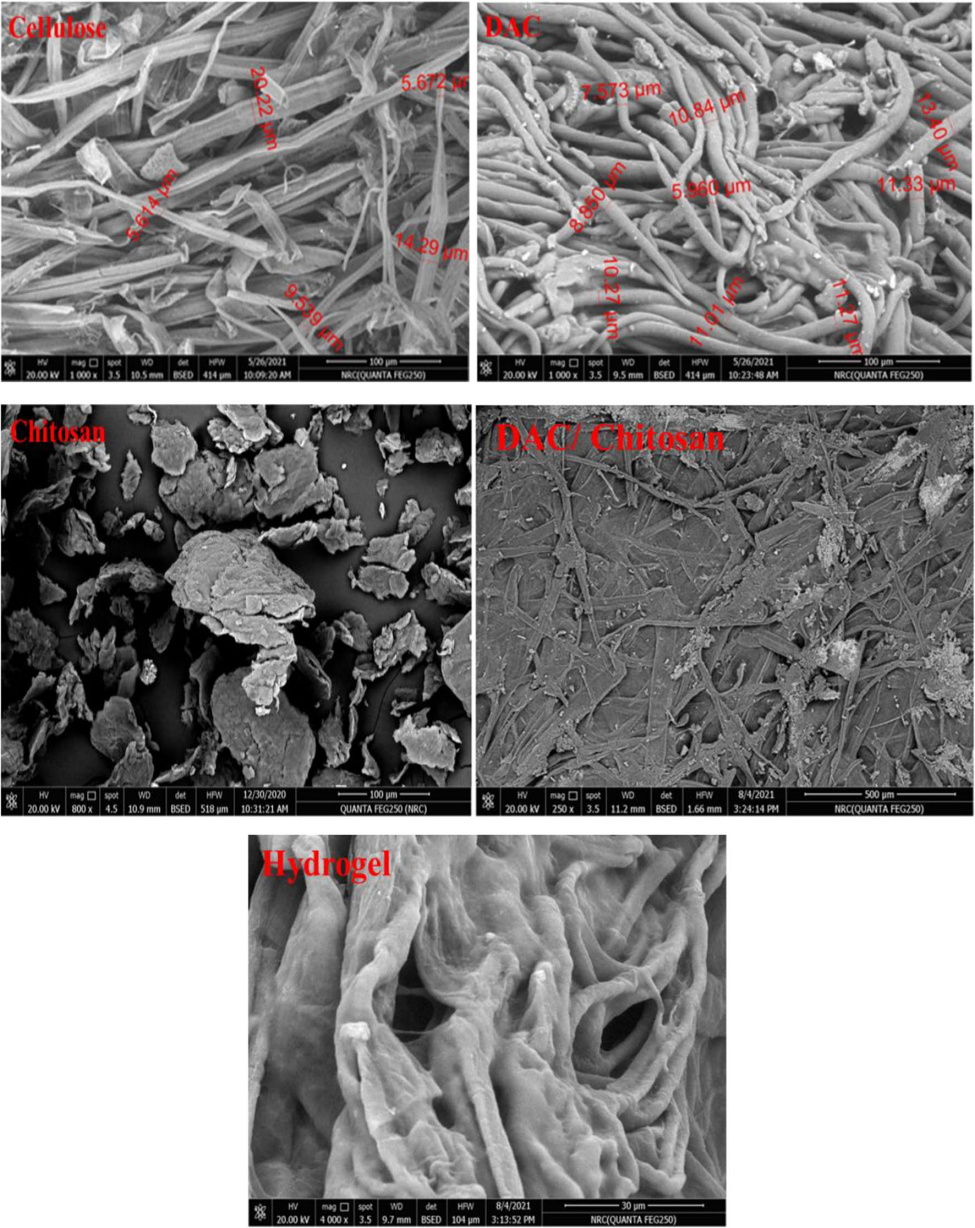
SEM of cellulose, DAC, chitosan, DAC/chitosan, and grafted DAC/chitosan

**Fig. 4:**
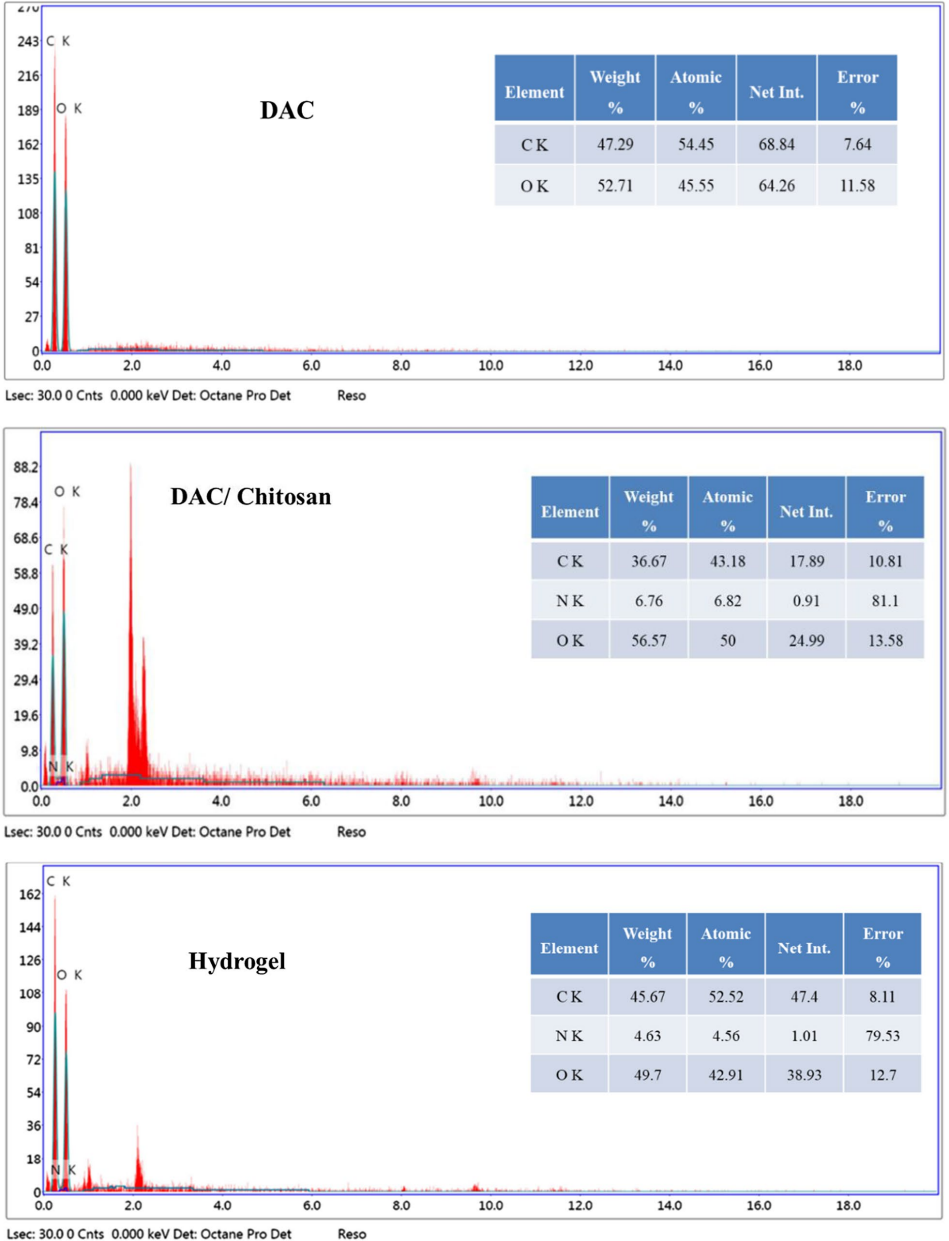
EDX analysis of; DAC, DAC/chitosan, and grafted DAC/chitosan

Figure 3 displays the SEM micrographs of the obtained cellulose fibers, chitosan, DAC, DAC/chitosan, and the obtained superabsorbent after the grafting polymerization of acrylic acid. Successful preparation of DAC with a diameter range from 5 to 15 µm and a length of several hundred micrometers is ensured. The corresponding EDX profile for these fibers revealed that carbon (47.29%) and oxygen (52.71%) are the major components of these structures. On the other hand, the chitosan appeared in around 8-µm thick flakes with a diameter of 95–120 µm. After chemical coupling between DAC and chitosan, the surface of the fibers became smoother, indicating that the ongoing reaction has proceeded via coating with the chitosan, which is additionally proved by the increased thickness indicating the chitosan, while in the soluble state accumulated on the surface of DAC Fibers. This was further confirmed by the relevant EDX (Fig. 4), from which it is apparent that a marked change in the components’ percentages took place; the carbon level dropped to 36.67%, and the oxygen level changed to 56.6%, whereas the nitrogen element has emerged with a level of 6.76%, which ensures the chemical linking between chitosan and DAC. After copolymerization on this hybridized backbone with acrylic acid, the corresponding micrograph of the obtained superabsorbent shows a porous structure. The relevant EDX analysis on the superabsorbent demonstrated a drop of the nitrogen content to 4.63%, which proves the graft copolymerization reaction. The superabsorbent structure is robust, while the hybridized fibers constitute the supporting pillars of this structure. This corroborates the growing of the graft polymer chains from the surface of the fibers, and the interactions between the chains are responsible for the observed mechanical integrity.

### Swelling studies of hydrogels

**Fig. 5:**
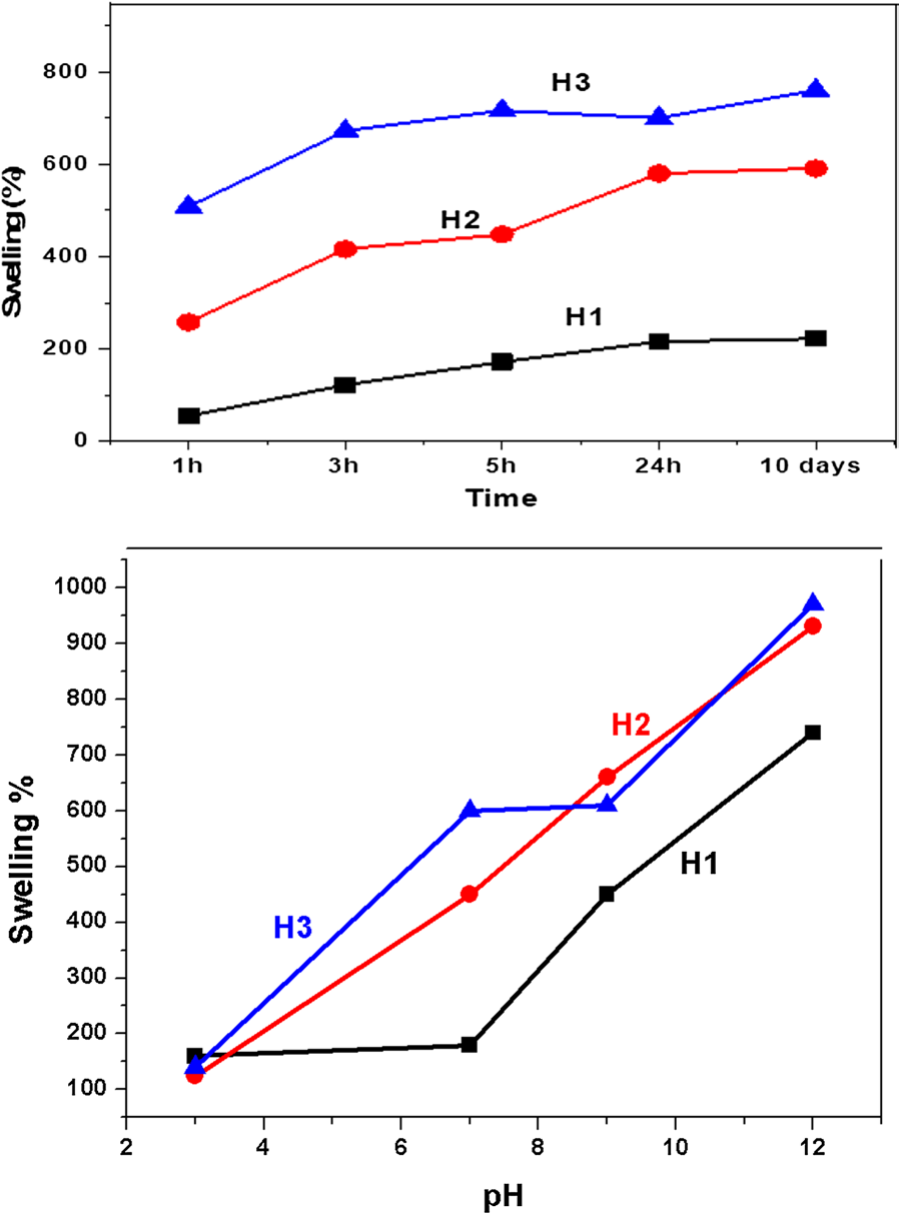
Effect of time and pH on the swellability of the grafted DAC/chitosan

Increasing the feed monomer in grafting polymerization is generally approved to increase the grafted amount. The swelling of the hybrid structures was studied as a function of time (1 h 10 days) and pH (3–12) (Fig. 5). All structures followed the same trend: the water absorption level continuously increased with time and pH. Figure 5 indicates that the amount of grafted acrylic acid in the DAC/chitosan hybrid backbone significantly impacts water absorption with time. H3 acquired the highest water absorption (872%) within 3 h of immersion in water. Thus, the samples can be sorted according to their water absorption level at equilibrium in the order H3 > H2 > H1, with 591% and 222% maximum absorption levels for H2 and H1, respectively.

Figure 5 shows the effect of pH on the swellability of hydrogels, and it was found that H3 exhibited the highest absorption level over the whole pH range, followed by H2. At the same time, H1 acquired the lowest swelling value among these structures. At pH 12, the swelling ratio reached 950% for H3, 900% for H2, and declined to 700% for H1. The least water absorption was observed at pH 3, where all structures acquired a close value of around 150%. The hybrid structures swelled systematically over the pH range from 3 to 12, with the superiority always in the H3 > H2 > H1 order. It is clear from the effect of time and pH on the hydrogel swellability that H3 has maximum absorption levels over H1 and H2. This was motivating to use H3 in a further study to remove heavy metal ions from water.

### Sorption study

**Fig. 6:**
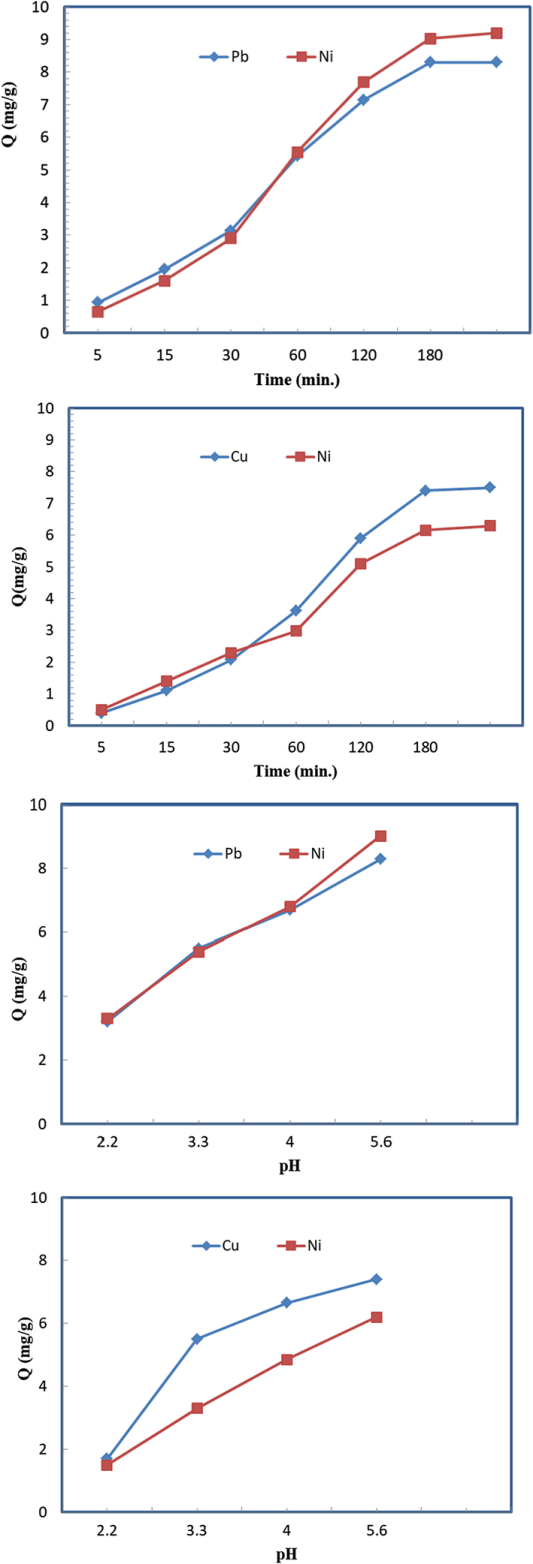
Effect of contact time and pH on the adsorption of binary solution comprising 10 mg/L of (Pb^2+^, Ni^2+^) and (Ni^2+^, Cu^2+^) on the grafted DAC/chitosan using adsorbent dose of 1 g/L over contact time of 120 min, at pH ≈ 5.6, and room temperature

In its pure form, cellulose is a weak adsorbent to metal ions, showing low adsorption capacity, which is attributed to the low activity of the hydroxyl groups. For that, the introduction of versatile, functional groups with high activity, such as nitrile, carboxyl, amine, sulfur, and amino groups, have been explained to improve the activity of cellulosic materials to act as ligands for metal ions, which leads to an increase the adsorption capacity [45]. Furthermore, the arrangement and distribution of the inserted functional groups in a tighter space as a result of the hybridization of functionalized cellulose and chitosan, followed by grafting of polyacrylic acid chains, which was confirmed by several techniques, provides a suitable environment for selective coordination with metal ions on a competitive basis in the case in a group of metal ions are present. Therefore, it was initially essential to study the adsorption of different elements, such as Pb^2+^, Ni^2+^, and Cu^2+^, when they are present as binary mixtures. Thus, the graft copolymer was left in prolonged contact time with binary mixtures of (Pb^2+^, Ni^2+^) and (Ni^2+^, Cu^2+^) until equilibrium was achieved, as displayed in Fig. 6. Unfortunately, clear discrimination between these elements was very hard, except at the beginning of 60 min for the binary mixture (Pb^2+^, Ni^2+^), which means that the graft copolymer shows similar affinity to such elements under these conditions up to 90 min for the mixtures. After that, the adsorption equilibrium was reached in 150–180 min.

On the other hand, the pH of aqueous solutions is one of the most important controlling parameters affecting the adsorbent efficiency for removing metal ions. The impact of pH on the adsorption of (Pb^+2^, Ni^+2^) and (Ni^+2^, Cu^+2^) binary mixtures using the prepared adsorbent was investigated as a function of pH in the range 2.2–5.6. We could not go farther in this range because OH could precipitate these metal ions to form metal (II) hydroxide above pH 6. The results are depicted for the ion mixtures in Fig. 6. The extractability of the ions from the solution increased with the elevation of the pH from 2.2 to 5.6, which can be attributed to the solubility enhancement of the metal ions and the degree of ionization of the adsorbent during removal. The maximum removal efficiencies of (Pb^+2^, Ni^+2^) and (Cu^+2^, Ni^+2^) reached (80.6, 79.9%) and (60.2, 64.4%), respectively. Under strongly acidic conditions, the adsorbent surface is entirely protonated with hydronium ions, which strongly oppose metal ions adsorption on the active sites**.** However, the separately of the adsorbent for Pb^+2^ and Ni^+2^ was very weak, especially at pH values of 2.2 and 5.6. On the contrary, for the mixture of Cu^+2^, and Ni^+2^, the separately is enhanced at pH between 3.3 and 4 while diminished at 2.2 or 5.6.

### Adsorption kinetics

The kinetic investigations of adsorption of the binary mixtures of the elements, as mentioned above, were studied to enable prediction of the rate at which these metal elements can be removed from aqueous solutions and provide an understanding of the adsorption mechanism. Different known kinetic models were employed for such a purpose.

#### Pseudo-first-order

The rate of metal ions adsorption on an adsorbent is based on its capacity to accommodate such ions. The pseudo-first-order model is applied to determine the mass transfer coefficient [46]. The integral form of the pseudo-first-order model can be generally expressed as follows:

$$\mathrm{log }\left(\mathrm{qe }-\mathrm{ qt}\right)=\mathrm{log qe}-\frac{\mathrm{k}1,\mathrm{ads}}{ 2.303} X t$$

where q_e_ and q_t_ are the amounts of metal ions adsorbed(mg/g) at equilibrium and at time t (min), respectively, while k_1_(1/min) is the rate constant of first-order adsorption.

**Fig. 7:**
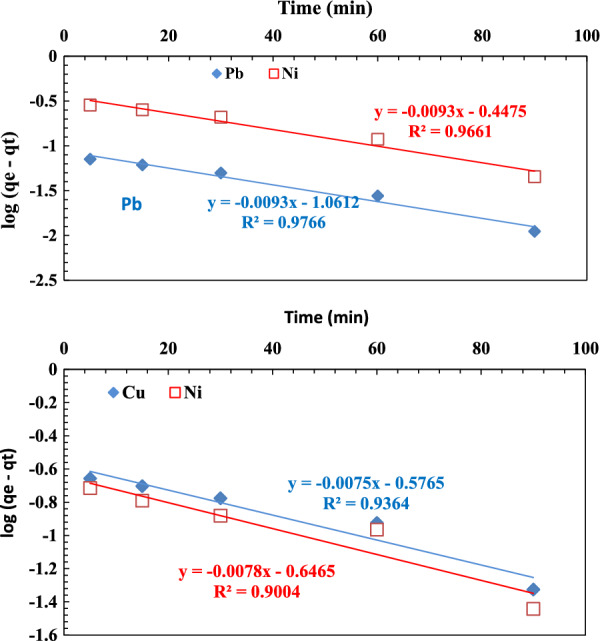
Pseudo-first-order plot for the adsorption of the binary solution (Pb^2+^, Ni^2+^) and (Cu^2+^, Ni^2+^) onto the grafted DAC/chitosan (pH ≈ 5.6, concentration of metal ions 10 mg/L, adsorbent dose 1 g/L)

Plotting of log (q_e_–q_t_) versus t for the binary ions mixtures of (Pb^2+^, Ni^2+^) and (Ni^2+^, Cu^2+^) are presented in **(**Fig. 7**).** The corresponding R^2^ values are 0.977, 0.966 for (Pb^2+^, Ni^2+^) and 0.9, 0.936 for (Ni^2+^, Cu^2+^), respectively, indicating good conformity between the experimental and calculated q_e_ values, which reflects the excellent applicability of this model to describe the adsorption process of (Pb^2+^, Ni^2+^) and (Ni^+2^, Cu^2+^) ions onto the graft DAC/chitosan.

#### Pseudo-second-order

The pseudo-second-order kinetic is developed to express the adsorption system of divalent metal ions onto an adsorbent [47]. The following equation can express the second-order kinetics:

$$\mathrm{t}/\mathrm{qt}=\frac{1}{(\mathrm{k}2,\mathrm{ads }\times \mathrm{qe})2}+\frac{t}{qe}$$

where k_2_ (g/mg min) is the rate constant of second-order adsorption.

**Fig. 8:**
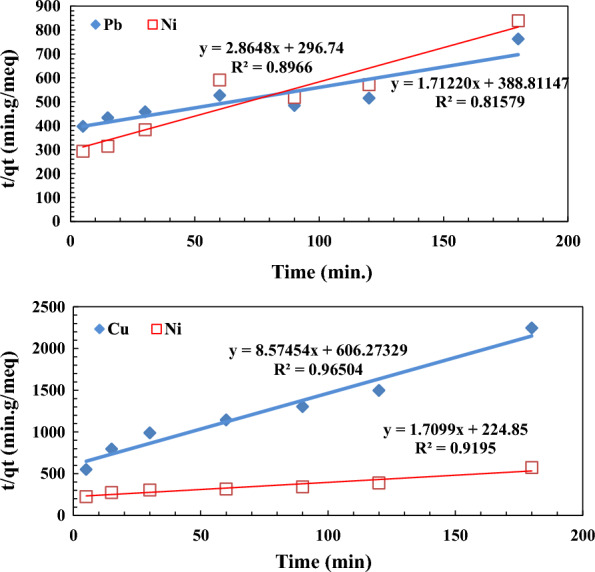
Pseudo-second-order plot for binary solution of (Cu^2+^, Ni^2+^) and (Pb^2+^, Ni^2+^) adsorption onto the grafted DAC/chitosan (pH ≈5.6, concentration of metal ions 10 mg/L, adsorbent dose 1 g/L)

A linear plot of t/qt versus t is shown in Fig. 8 for the binary solution (Cu^2+^, Ni^2+^) with a relatively low R^2^. In contrast, the experimental q_e_ values disagree with the calculated values obtained from the linear plots.

On the other hand, in the case of the binary solution (Pb^2+^, Ni^2+^), the obtained R^2^ values from the corresponding plots (Fig. 8) were satisfactory high (0.965, 0.919 for Pb^2+^, Ni^2+^, respectively). However, the experimental q_e_ values did not agree with the calculated values obtained from the linear plots.

These data reveal the second-order kinetic model could be better for describing the adsorption process of (Cu^2+^, Ni^2+^) on the graft copolymer whereas for the binary mixture (Pb^2+^, Ni^2+^) the first-order kinetic model prevail.

### Adsorption isotherms

Adsorption isotherms present parameters that describe the affinity of an adsorbent towards specific adsorbates such as metal ions. Investigation of the performance of a polymeric adsorbent is significant for getting a precise equilibrium correlation between the solid and liquid-phase concentrations of metal ions [48]. Thus, it is essential to find the corresponding equilibrium data for removing of metal ions such as (Pb^2+^, Ni^2+^) and (Cu^2+^, Ni^2+^) using the developed graft copolymer with different isotherm models.

#### Langmuir isotherm

The Langmuir isotherm model [49] has been widely used innumerous processes of metal ions removal via adsorption. It is based on the assumption that metal ions uptake proceeds on a homogeneous surface via monolayer adsorption without any interaction between adsorbed metal ions, considering that all adsorption sites have equal activity. In contrast, the adsorption at one site will not affect the adsorption at a neighboring site. Accordingly, the Langmuir isotherm is best applicable for monolayer adsorption onto a surface containing a finite number of identical sites. It can be expressed by the following equation:

$$ {\text{C}}_{\text{e}} /{\text{q}}_{\text{e}} \, = \,{1}/\left( {{\text{K}}\, \times \,{\text{q}}_{{\text{max}}} } \right)\, + \,{\text{C}}_{\text{e}} /{\text{q}}_{{\text{max}}} $$

where C_e_ is the concentration at equilibrium (mg/L), q_e_ is the adsorbed amount at equilibrium (mg/g), q_max_ is the maximum adsorption capacity corresponding to the complete monolayer coverage (mg/g), and K is the Langmuir constant related to the adsorption energy.

**Fig. 9:**
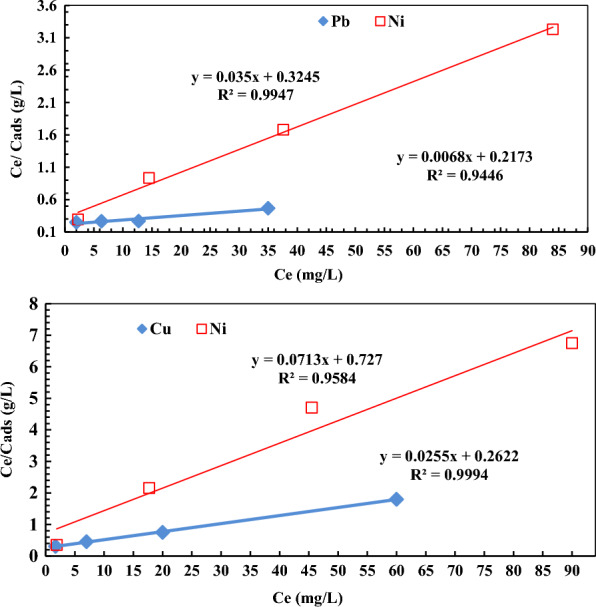
Langmuir model isotherm for binary solution of (Pb^2+^, Ni^2+^) and (Cu^2+^, Ni^2+^) adsorption onto the grafted DAC/chitosan after contact for 120 min using adsorbent dose of 1 g/L at pH ≈ 5.6)

A linear plot of Langmuir adsorption isotherm (C_e_/q_e_ vs. C_e_) demonstrates the validity of Langmuir isotherm to follow the adsorption of metal ions as revealed in Fig. 9. The values of q_max_ and K for (Pb^2+^, Ni^2+^) and (Cu^2+^, Ni^2+^) ions were calculated from the slope and the intercept of the linear plots C_e_/q_e_ versus C_e_. The results are given in Table 1, which showed a good fit was achieved for the data with the Langmuir isotherm model. Further, the Langmuir parameters can also be employed to deduce the affinity between the different metal ions and the grafted DAC/chitosan as adsorbent using a dimensionless separation factor (R_L_), which can be defined from the following equation [49]:

$$ {\text{R}}_{\text{L}} \, = \,{1}/{1}\, + \,({\text{K C}}_{\text{o}} ) $$

**Table 1:** The separation factor for the binary solutions of (Pb^2+^, Ni^2+^) and (Cu^2+^, Ni^2+^) adsorption on the grafted DAC/chitosan

C_o_ (mg/L)	(Pb^2+^, Ni^2+^)		(Cu^2+^, Ni^2+^)	
	R_L_ of Pb^2+^	R_L_ of Ni^2+^	R_L_ of Ni^2+^	R_L_ of Cu^2+^
10	0.0152	0.0156	0.017	0.0159
30	0.0051	0.00524	0.00569	0.00531
60	0.00243	0.0026	0.00285	0.00265
110	0.00132	0.00143	0.00158	0.0015

where K is the Langmuir constant (L/mg) and C_o_ is the concentration of metal ions, mg/L. The R_L_ value indicates whether the adsorption is unfavorable (R_L_ > 1), (R_L_ = 1) or favorable (0 < R_L_ < 1), or irreversible (R_L_ = 0).

Figure 9 shows that the adsorption of (Pb^+2^, Ni^+2^) and (Cu^+2^, Ni^+2^) increased in parallel with a concentration of metal ions. In addition, the obtained R_L_ values for these metal ions` adsorption indicate that adsorption is favorable even at higher concentrations of metal ions (Table 1).

#### Freundlich isotherm

The Freundlich isotherm theory [50] dictates that the ratio of adsorbate amount to a given mass of adsorbent concerning the adsorbate concentration in a solution is not constant at different concentrations. The magnitude of adsorption heat decreases with increasing the extent of adsorption. The Freundlich isotherm adsorption model indicates an adsorbent's surface heterogeneity, while it can be expressed by the following equation**:**

$$\mathrm{log }{q}_{e}=\mathrm{log}{K}_{f}+\frac{1}{n} log{C}_{e}$$

where K_F_ and 1/n are constants ascribed to capacity and intensity of adsorption process, respectively.

**Fig. 10:**
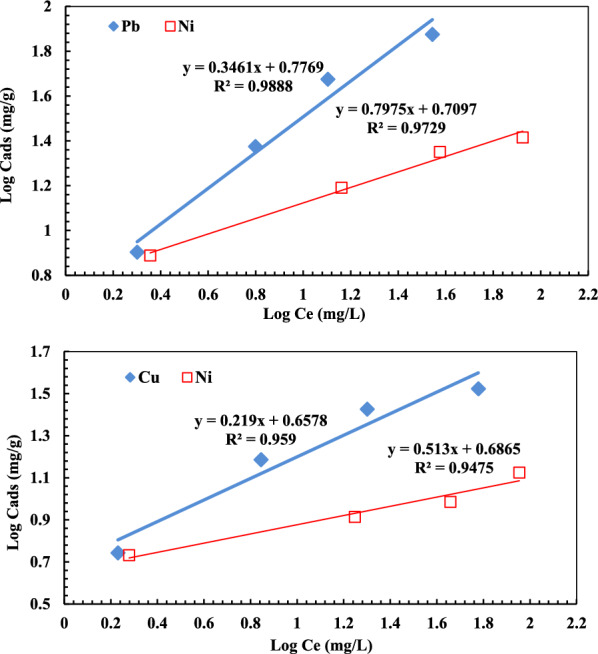
Freundlich isotherm for binary solution of (Pb^2+^, Ni^2+^) and (Cu^2+^, Ni^2+^) adsorption onto the grafted DAC/chitosan after contact for 120 min using adsorbent dose of 1 g/L at pH ≈ 5.6

The values of n and K_F_ for (Pb^2+^, Ni^2+^) and (Cu^2+^, Ni^2+^) ions were obtained from the slope and the intercept of linear plots between log q_e_ versus log C_e_ (Fig. 10) while the related correlation coefficients (R^2^) are presented in Tables 2. Interestingly, the R^2^ values confirmed that the developed grafted DAC/chitosan exhibits good fit with both Langmuir and Freundlich isotherms.

**Table 2:** Summary of isotherm models parameters for (Pb^2+^, Ni^2+^) and (Cu^2+^, Ni^2+^) adsorption onto the grafted DAC/chitosan

Binary mixture	Metal ion	Langmuir model			Freundlich model		
		K L/mg	q_max_ (mg/g)	R^2^	K_f_	n	R^2^
(Pb^2+^/ Ni^2+^)	Ni^2+^	6.3	28.57	0.995	5.98	2.89	0.9888
	Pb^2+^	6.49	147.06	0.9446	5.13	1.254	0.9729
(Cu^2+^/ Ni^2+^)	Ni^2+^	5.76	14.03	0.9584	4.55	4.57	0.959
	Cu^2+^	6.18	39.22	0.999	4.86	1.95	0.948

### Selective removal of metal ions by grafted DAC/chitosan

**Fig. 11:**
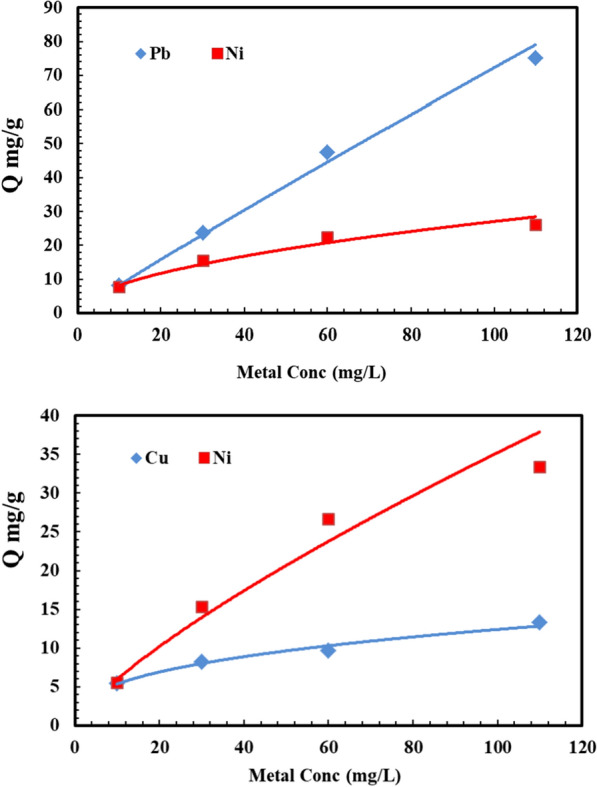
Effect of initial ions concentration of Pb^2+^, Ni^2+^, and Cu^2+^ in a binary mixture of (Pb^2+^, Ni^2+^) and (Cu^2+^, Ni^2+^) on the adsorption into the grafted DAC/chitosan at operating conditions (contact time 120 min, adsorbent dose1 g/L at pH ≈ 5.6)

The adsorption activity of the prepared adsorbent (grafted DAC/chitosan) was investigated towards metal ions existing together at equal initial concentrations. This was undertaken at different concentrations in the 10–110 mg/L range of each ion while the pH was kept constant at 5.6, as displayed in Fig. 11.

Generally, it can be observed that the removal of the grafted DAC/chitosan towards metal ions is increased to 68.1, 23.6% for the combination of Pb^2+^ and Ni^2+^, respectively, and 45.5 and 18.2% for the combination Cu^2+^ and Ni^2+^ when the metal ions concentration is increasing from 10 to 110 mg/L. This phenomenon is most likely due to the saturation of the active sites of the adsorbent with the metal ions at higher concentrations, keeping in mind that the preference for Pb^2+^/ Cu^2+^ against Ni^2+^ is predominant for all combinations. Interestingly, a selective removal and more effective separation of the metal ions from each other can be achieved at higher concentrations. In contrast, this trend appears more evident in the case of Pb^2+^/ Ni^2+^ than in the other systems. This behavior is presumably related to a minor extent to the bigger size of Ni^2+^ concerning other ions and, to a significant degree, to the nature of the polymeric material, which can be supported by the high removal capacity of these ions without competitive disturbance from the present Ni^2+^. More interestingly, the removal of Ni^2+^ is still relatively superior with respect to other comparable materials, even in the presence of other competing ions [51].

#### Desorption studies

The ability to desorb metal ions from an adsorbent is an important factor in determining the lifetime of the adsorbent. Thus, regenerability of hydrogel was investigated. The results of the performance of the sorbents for the removal of metal ions from binary mixtures are collected in Tables 3, where it can be emphasized that the sorbent was very active for the removal of the ions during the second and third cycles and maintained 92.6 and 86.4% compared with the original activity of sorbent during the first cycle, respectively, in case of Pb^2+^. In contrast, in the case of Ni^2+^, the sorbent retained 99.9 and 84.4% for the second and third cycles compared with the first one.

**Table 3:** Repetitive cycles for removal of Pb^2+^, Ni^2+^, and Cu^2+^ from their binary mixtures at optimized operating conditions

Mixture →	(Pb^2+^/ Ni^2+^)		(Cu^2+^/ Ni^2+^)	
Cycle ↓	Pb^+2^ Q (mg/g)	Ni^+2^ Q (mg/g)	Cu^2+^ Q (mg/g)	Ni^2+^ Q (mg/g)
1	8.1	7.7	8.3	8.1
2	7.5	7	7.9	7.6
3	7	6.5	7.4	7.4

The sorbent behaved very similarly in the case of the binary mixture of Cu^2+^ and Ni^2+^, where the activity of the sorbent to remove Cu^2+^ in the second cycle was 95.2% concerning its activity during the first cycle, declined to 89.2% during the third one. On the other hand, in the case of Ni^2+^, the retained activity in the second and third cycles reached 93.8 and 91.4%, respectively.

## Conclusions

Cellulose can be functionalized via oxidation to gain high content of aldehyde groups on its surface. The aldehyde-functionalized cellulose can be coupled with chitosan to yield a chemically active backbone with improved handling and high surface area, making it a viable backbone for grafting copolymerization with acrylic acid. This has driven the resulting highly versatile structure to acquire a porous network incorporating pores of various size distributions. The functionalization with copious hydrophilic groups and complicated mesh structure are deemed the driving force for elevated swelling in aqueous media. Such characteristics can qualify this novel material to act as an active adsorbent for heavy metal ions on a competitive basis from their aqueous mixtures. The high swelling capacity of the adsorbent (872%) was referred to as the developed high porosity with various size distributions as watched by SEM. A selective adsorption trend for efficient removal of Ni^2+^can be achieved, even though other competing ions of smaller size, such as Pb^2+^and Cu^2+^, are present. The extensive crowding of polymeric chains with various functional groups distributed over pores with multiple-size distributions is responsible for such selectivity. The kinetic study showed the adsorption of Pb^2+^, Ni^2+^, and Cu^2+^ in a binary mixture of (Pb^2+^, Ni^2+^) and (Cu^2+^, Ni^2+^) onto the grafted DAC/chitosan fit with both Langmuir and Freundlich isotherms. In addition, the grafted DAC/chitosan hydrogel exhibited excellent reusability. This study provided a highly efficient bioadsorbent for the removal of heavy metals from an aqueous solution.

## Data Availability

All data and materials are available.
